# Supplementary material for: Associations of Atrial Fibrillation with Mild Cognitive Impairment and Dementia: An Investigation Using SPRINT Research Materials
Source: J Clin Med. 2022 Sep 30;11(19):5800. doi: 10.3390/jcm11195800 (PMC9572519; doi:10.3390/jcm11195800)
Supplement: Supplementary file 1 [file jcm-11-05800-s001.zip › jcm-1908957-supplementary.pdf]

Supplemental Table S1. Hazard ratios and 95% confidence intervals for association of 4 levels of (time-dependent) AF status with dementia or MCI, stratified by education.

	No AF	Self-report only AF	Safety event AF only or self-report + safety events	ECG AF
**No College (N = 2,718)**				
**N. cases**	350	15	16	24
**P-years**	10,602	523	316	355
**Incidence rate***	33.0	28.7	50.6	67.6
**Model 1**	1 (Ref.)	0.72 (0.46, 1.12)	1.00 (0.54, 1.86)	2.17 (1.24, 3.81)
**Model 2**	1 (Ref.)	0.71 (0.44, 1.14)	0.97 (0.52, 1.80)	2.12 (1.19, 3.78)
**College (N = 5,751)**				
**N. cases**	388	25	12	28
**P-years**	24,114	1,512	638	948
**Incidence rate***	16.1	16.5	18.8	29.5
**Model 1**	1 (Ref.)	0.73 (0.51, 1.04)	0.99 (0.55, 1.80)	1.18 (0.68, 2.04)
**Model 2**	1 (Ref.)	0.76 (0.52, 1.11)	0.99 (0.54, 1.79)	1.19 (0.68, 2.10)
**P-value for interaction**		0.24	0.76	0.84

*Crude incidence rate, per 1000 p-years. Model 1: adjusted for age at randomization, sex, and race/ethnicity Model 2: adjusted for age at randomization, sex, race/ethnicity, OAC use, smoking, history of CVD, total cholesterol, HDL cholesterol, eGFR, systolic and diastolic pressure, and BMI.

Supplemental Table S2. Hazard ratios and 95% confidence intervals for association of 4 levels of (time-dependent) AF status with dementia or MCI, stratified by sex.

	No AF	Self-report only AF	Safety event AF only or self-report + safety events	ECG AF
		**Male (N = 5,503)**		
**N. cases**	468	26	19	39
**P-years**	22,268	1,420	617	989
**Incidence rate***	21.0	18.3	30.8	39.4
**Model 1**	1 (Ref.)	0.65 (0.46, 0.92)	0.87 (0.50, 1.50)	1.78 (1.12, 2.83)
**Model 2**	1 (Ref.)	0.67 (0.46, 0.98)	0.86 (0.50, 1.49)	1.77 (1.09, 2.88)
		**Female (N = 2,966)**		
**N. cases**	270	14	9	13
**P-years**	12,448	615	337	314
**Incidence rate***	21.7	22.8	26.7	41.4
**Model 1**	1 (Ref.)	0.98 (0.31, 1.58)	1.34 (0.67, 2.66)	1.23 (0.58, 2.61)
**Model 2**	1 (Ref.)	0.95 (0.58, 1.58)	1.24 (0.62, 2.46)	1.21 (0.56, 2.58)
**P-value for interaction**		0.43	0.46	0.38

*Crude incidence rate, per 1000 p-years. Model 1: adjusted for age at randomization, education, and race/ethnicity Model 2: adjusted for age at randomization, education, race/ethnicity, OAC use, smoking, history of CVD, total cholesterol, HDL cholesterol, eGFR, systolic and diastolic pressure, and BMI.

Supplemental Table S3. Hazard ratios and 95% confidence intervals for association of 4 levels of (time-dependent) AF status with dementia or MCI, stratified by race*.

	No AF	Self-report only AF	Safety event AF only or self-report + safety events	ECG AF
		**White (N = 4,949)**		
**N. cases**	356	25	20	35
**P-years**	19,985	1,504	773	1,078
**Incidence rate****	17.8	16.6	25.9	32.5
**Model 1**	1 (Ref.)	0.61 (0.43, 0.87)	0.95 (0.57, 1.60)	1.39 (0.86, 2.26)
**Model 2**	1 (Ref.)	0.61 (0.42, 0.90)	0.97 (0.58, 1.63)	1.36 (0.83, 2.25)
		**Black (N = 2,492)**		
**N. cases**	260	11	6	7
**P-years**	10,465	391	118	140
**Incidence rate****	24.8	28.1	50.8	50.0
**Model 1**	1 (Ref.)	1.05 (0.61, 1.81)	1.72 (0.76, 3.92)	1.24 (0.49, 3.16)
**Model 2**	1 (Ref.)	1.12 (0.64, 1.96)	1.53 (0.67, 3.52)	1.30 (0.51, 3.31)
**P-value for interaction**		0.12	0.47	0.69

*Hispanic and other race/ethnicities not included in this analysis due to low AF counts. **Crude incidence rate, per 1000 p-years. Model 1: adjusted for age at randomization, education, and sex Model 2: adjusted for age at randomization, education, sex, OAC use, smoking, history of CVD, total cholesterol, HDL cholesterol, eGFR, systolic and diastolic pressure, and BMI.
